# Perceptions of distinctions between patient and healthcare zones among intensive care unit nurses at a Korean tertiary hospital: A cross-sectional study

**DOI:** 10.1371/journal.pone.0311298

**Published:** 2024-11-15

**Authors:** Hayoung Chang, JaHyun Kang

**Affiliations:** 1 College of Nursing, Seoul National University, Seoul, Republic of Korea; 2 Research Institute of Nursing Science, Seoul National University, Seoul, Republic of Korea; Center for Primary Care and Public Health: Unisante, SWITZERLAND

## Abstract

**Background:**

Intensive care unit (ICU) patients face higher infection risks from invasive procedures, highlighting the critical role of ICU nurses in infection prevention. Clear differentiation between the patient and healthcare zones is essential for effective hand hygiene and disinfection, yet research on this topic is limited.

**Aim:**

To assess ICU nurses’ perception of the concept of patient and healthcare zones and evaluate their similarity and accuracy in identifying the zones.

**Methods:**

A descriptive survey was conducted at a 2,732-bed tertiary hospital in Korea from 28 July to 27 August 2022. Participants were recruited from various ICUs through flyers. 225 questionnaires—with illustrations 27 item locations for three ICU scenarios—were made available at nursing stations for voluntary completion. Participants were asked to classify items into the patient zone or the healthcare zone. Similarity scores reflected participant agreement, while accuracy scores measured the proportion of correct answers. Participants’ free-text opinions regarding zone classification were analysed thematically.

**Findings:**

104 nurses participated voluntarily. Average similarity and accuracy were 84.7% and 82.7%, respectively. The top 8 items, with over 97% similarity and accuracy, were all frequently in contact with ICU patients (e.g., pulse oximeter, Levin tube, central line, urine bag, and patient bed). The bottom 7 items, with less than 80%, included the glucometer, flashlight, trolley, and sink. Participants with higher education levels had significantly higher similarity (*p* = .044) and accuracy (*p* = .033), whereas those already familiar with the patient-zone concept had significantly higher accuracy (*p* = .009). From the free-text analysis, participants considered factors beyond proximity to the patient, such as patient contact, room type, and distance.

**Conclusions:**

To address zone classification challenges, clear criteria for patient and healthcare zones, space redesign, and educational programs are recommended. Further research is necessary to improve greater clarity and consensus regarding patient and healthcare zones to enhance infection control practices.

## Introduction

The European Centre for Disease Prevention and Control (ECDC) estimated 4.5 million healthcare-associated infection (HAI) episodes from 2016 to 2017 in acute care hospitals in the European Union (EU) and European Economic Area (EEA) [1]. Indeed, healthcare environments have been underscored as potential reservoirs for pathogens that significantly transmit HAIs [2–16]. Colonised or infected patients can shed pathogens into their surroundings, and these microorganisms can survive for extended periods on items and surfaces [3].

To prevent pathogen transmission and reduce HAIs, the World Health Organization introduced the ‘Five Moments for Hand Hygiene’ model, which has been adopted globally for training, monitoring, and reporting among healthcare personnel (HCP) across various healthcare settings [17, 18]. This model incorporates the concept of two distinct zones within the healthcare environment: patient and healthcare [17]. The patient zone revolves around patient X and their immediate surroundings, encompassing the patient’s intact skin and all inanimate surfaces directly or indirectly contacted by the patient [17]. To prevent the transmission of HAI pathogens, all items entering and leaving the patient zone should be decontaminated [17, 18]. Conversely, the healthcare zone includes all surfaces outside the patient’s designated area [17].

Nevertheless, the complex nature of ongoing tasks, resource constraints, and the diversity of settings have generated confusion among HCP regarding the proper indication of hand hygiene timing and the clear differentiation of zones [19–24]. In healthcare settings, a lack of congruent perception regarding patient zones, which clarifies the indications for hand hygiene, may result in unintentional pathogen transmission [25].

Intensive care unit (ICU) patients are at a higher risk of developing HAIs owing to their severe conditions and exposure to invasive devices [26]. In the EU and EEA, the ECDC estimated the prevalence of patients with at least one HAI in ICUs as 19.2%, the highest number compared with all other specialties combined (5.2%) [1]. As nurses typically have the most extensive direct contact with patients in hospitals [27], ICU nurses play a crucial role in infection control. A study involving 200 ICU nurses at a tertiary hospital in Korea reported that 72% of participants identified ‘after touching a patient’s surroundings’ as being the most ambiguous moment for hand hygiene [22]. Among these respondents, 41% attributed their ambiguity to the challenge of distinguishing the patient zone from the healthcare zone [22]. Another study conducted in a general ward in Switzerland reported limited accuracy (68%) and similarity (77%) among doctors and nurses who were tasked with classifying 32 item cards into either patient or healthcare zones [24].

Given the limited prior research on this topic and the importance of HCP’s zone perceptions for infection control, we aimed to examine how the zone concept is applied in daily care activities by ICU nurses and obtain insights for improving infection control practices like hand hygiene or item disinfection. The study objectives were to assess ICU nurses’ perceptions of the zone concept and evaluate their accuracy and similarity in identifying the zones by presenting scenarios and illustrations depicting various patient care situations with item use.

## Methods

This descriptive survey was conducted at a 2,732-bed tertiary hospital in Korea, from 28 July to 27 August 2022. Ethics approval was obtained from the Asan Medical Center Institutional Review Board. Nurses with at least 2 months of independent work experience providing direct patient care within ICUs were eligible for this study. Nurse administrators who did not engage in direct patient care were also excluded. Participants were recruited from various ICUs—medical, surgical, neurological, neurosurgical, and paediatric—through flyers posted at nursing stations. A minimum of 180 participants was needed to analyse significant differences based on general characteristics. To account for a 20% dropout rate, the target sample size was set at 225 participants. Accordingly, 225 printed consent forms and questionnaires were made available at the ICU nursing stations, allowing nurses to access and complete them voluntarily. Written informed consent was collected from all participants.

**Fig 1:**
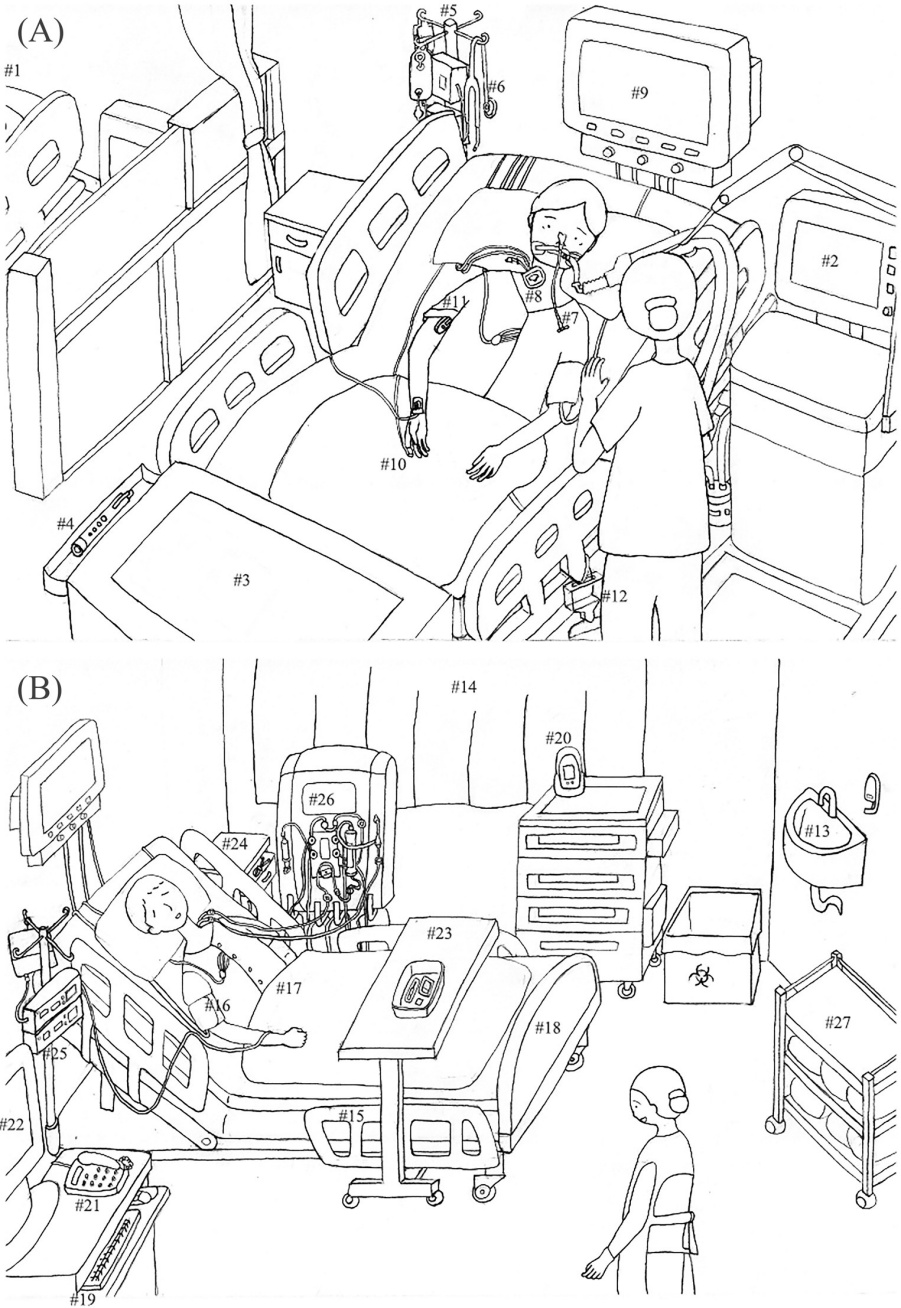
Illustrations of the three nursing care scenarios. (A) shows the locations of items in Scenario 1, which depicts open space. (B) shows the locations of items in Scenario 2 and 3, which depicts a private room.

The study questionnaire developed for this study comprised basic demographics, a main survey, and additional opinions (see S1 File, the English version of the questionnaire). Definitions of patient and healthcare zones were provided at the beginning of the questionnaire. First, the demographic questions included age, gender, work experience, department, education level, prior knowledge of patient zone concepts, and learning route. Second, for the main survey, 47 items associated with the risk of infection transmission were initially included in the literature review [8–16]. The researcher with ICU work experience created three scenarios depicting the use of these items: (1) assessing a patient’s condition and measuring vital signs; (2) patient bathing and measuring body weight; (3) measuring blood glucose levels and administering medication. These scenarios were chosen because they include routine care activities that ICU nurses commonly perform using the selected items. While the patient in Scenario 1 was situated in an open ICU area, the patient in Scenarios 2 and 3, who was the same individual, was placed in a private room. To prevent confusion among respondents regarding nursing care scenarios and item locations, the researcher created illustrations for three scenarios providing the locations of the 47 items. Content validity was assessed by five area experts, all of whom held a master’s degree or higher: one nursing professor with infection prevention and control (IPC) expertise, two IPC practitioners, and two ICU nurses with more than 5 years of experience. Items were rated as follows: 4 points for highly relevant, 3 points for somewhat relevant, 2 points for minimally relevant, and 1 point for not relevant. After eliminating 17 items with a content validity index of 0.75 or less and 3 items with similar characteristics, the final 27 items were included (Fig 1). Throughout the main survey, participants were tasked with categorising each item into the patient zone, healthcare zone, or ‘difficult to determine’ option with a brief description of their reason. Third, seven open-ended questions were posted to elicit the participants’ logic in decision-making. Following each scenario, three identical questions were presented, prompting participants to consider whether, if the situation changed, any items in the corresponding scenario might belong to the opposite zone compared to their initial selection and to explain the reason for the change. At the end of the survey, four additional open-ended questions were asked: (1) participants’ most significant criteria when categorising items into the patient or healthcare zone; (2) challenges encountered when differentiating between the two zones during regular work; (3) suggestions for addressing the identified challenges; (4) specific items that were difficult to categorise and confusing when determining the appropriate time for disinfection or hand hygiene.

For data analysis, the two IPC practitioners determined the correct answers for allocating the 27 items to their respective zones and subsequently validated them with a professor who has IPC expertise. For each item, the similarity among participants’ responses was calculated by measuring the proportion of each participant’s responses that matched the majority choices, and the accuracy was determined by calculating the proportion of correct responses that concurred with the consensus reached by the IPC practitioners and professor. Similarity and accuracy were calculated for each participant to investigate potential variations based on their general characteristics. In addition to descriptive statistical analyses, the Wilcoxon rank-sum test or Kruskal-Wallis test for discrete variables and Spearman’s rank correlation analysis for continuous variables were conducted using R version 4.0.2 (The R Foundation for Statistical Computing, Vienna, Austria). To delve into participants’ opinions, thematic analysis was undertaken to identify recurring themes and patterns within their free-text responses.

## Results

### Participants’ general characteristics

Of the 225 questionnaires distributed, 104 nurses completed the survey, resulting in a response rate of 46.2%. The participants’ average age was 29.0 years, with an average work experience of 5.9 years (Table 1). Most participants (88.5%) were aware of the patient zone concept, mainly from hospitals (78.8%).

**Table 1:** Characteristics of 104 survey participants.

Characteristics	Categories	No. (%)
**Age**	< 25	19 (18.3%)
	25 ≤ ~ < 30	47 (45.2%)
	30 ≤ ~ < 35	23 (22.1%)
	≥ 35	15 (14.4%)
**Gender**	Male	5 (4.8%)
	Female	99 (95.2%)
**Total clinical experience (years)**	< 1	6 (5.8%)
	1 ≤ ~ < 3	35 (33.7%)
	3 ≤ ~ < 5	20 (19.2%)
	5 ≤ ~ < 10	23 (22.1%)
	≥ 10	20 (19.2%)
**ICU experience (years)**	< 1	6 (5.8%)
	1 ≤ ~ < 3	38 (36.5%)
	3 ≤ ~ < 5	20 (19.2%)
	5 ≤ ~ < 10	21 (20.2%)
	≥ 10	19 (18.3%)
**Department**	MICU1	18 (17.3%)
	SICU1	11 (10.6%)
	NRICU	23 (22.1%)
	NSICU	8 (7.7%)
	PICU1	15 (14.4%)
	PICU2	29 (27.9%)
**Level of Education**	≤ Bachelor	95 (91.3%)
	≥ Master	9 (8.7%)
**Prior knowledge of patient zone concept**	No	11 (10.6%)
	Yes	92 (88.5%)
	No response	1 (1.0%)
**Learning route for the patient zone concept**	School only	10 (9.6%)
	Hospital only	66 (63.5%)
	School & Hospital	15 (14.4%)
	Hospital & Others	1 (1.0%)
	No response	1 (1.0%)
	Not applicable	11 (10.6%)

ICU, intensive care unit; SD, standard deviation; MICU, medical intensive care unit; SICU, surgical intensive care unit; NRICU, neurological intensive care unit; NSICU, neurosurgical intensive care unit; PICU, paediatric intensive care unit

### Zone allocation of the items

Regarding categorising 27 items into zones, participants allocated 20 (74.1%) to the patient zone and 7 to the healthcare zone (Table 2). Most participants’ responses regarding the curtain and glucometer diverged from the choices made by IPC practitioners and professors, indicating discrepancies in perspectives.

**Table 2:** Allocation results for the 27 items by the participants.

Scenario numbers (patient room type)	Items	Patient Zone N (%)	Healthcare Zone N (%)	Difficult to decide N (%)
**Scenario 1 (open ICU area)**	**#1 Another patient**	33 (31.7)	60 (57.7)	11 (10.6)
	**#2 Ventilator monitor**	87 (83.7)	17 (16.3)	0
	**#3 Patient cart**	56 (53.8)	48 (46.2)	0
	**#4 Flashlight**	75 (72.1)	28 (26.9)	1 (1)
	**#5 Pole**	93 (89.4)	11 (10.6)	0
	**#6 Stethoscope**	94 (90.4)	10 (9.6)	0
	**#7 Levin tube**	103 (99)	1 (1)	0
	**#8 Central line**	103 (99)	1 (1)	0
	**#9 Patient monitor**	89 (85.6)	15 (14.4)	0
	**#10 Pulse oximeter**	104 (100)	0	0
	**#11 Thermometer**	99 (95.2)	5 (4.8)	0
	**#12 Urine bag**	103 (99)	1 (1)	0
**Scenario 2 (single-patient room)**	**#13 Sink**	23 (22.1)	69 (66.3)	12 (11.5)
	**#14 Curtain**	50 (48.1)^a^	48 (46.2)	6 (5.8)
	**#15 Bed rail**	101 (97.1)	3 (2.9)	0
	**#16 Blood pressure cuff**	102 (98.1)	2 (1.9)	0
	**#17 Linen**	101 (97.1)	3 (2.9)	0
	**#18 Patient bed**	103 (99)	1 (1)	0
	**#19 Keyboard**	9 (8.7)	92 (88.5)	3 (2.9)
**Scenario 3 (single-patient room)**	**#20 Glucometer**	22 (21.2)	76 (73.1)^a^	6 (5.8)
	**#21 Telephone fixed beside computer**	6 (5.8)	94 (90.4)	4 (3.8)
	**#22 Computer**	8 (7.7)	93 (89.4)	3 (2.9)
	**#23 Bedside table**	95 (91.3)	9 (8.7)	0
	**#24 Tourniquet**	87 (83.7)	17 (16.3)	0
	**#25 Infusion pump**	87 (83.7)	16 (15.4)	1 (1)
	**#26 CRRT**	90 (86.5)	13 (12.5)	1 (1)
	**#27 Trolley**	27 (26)	73 (70.2)	4 (3.8)

^a^Majority answers are incorrect; ICU, intensive care unit; CRRT, continuous renal replacement therapy

### Similarity and accuracy in items’ zone allocation

When allocating zones for 27 items, the overall means for similarity and accuracy were 84.7% and 82.7%, respectively (Table 3). Among these, 12 items, which were either attached to the patient or were located nearby, showed more than 90% similarity and accuracy. For example, the pulse oximeter achieved 100% similarity and accuracy in allocation to the patient zone. However, among the seven items with similarity and accuracy lower than 74%, three (curtain, another patient, and sinks), which were located relatively distant from the patient, showed the lowest accuracy, ranging from 46.2% to 66.3%. Additionally, three other items (glucometers, flashlights, and trolleys [carts with wheels]) that were movable within the patient’s proximity ranged from 70.2% to 73.1%. Notably, a glucometer located on a patient cart, which was positioned away from the patient within a private room, showed 73.1% similarity and 21.2% accuracy, while a patient cart located immediately next to the foot of the bed in the open area of the ICU showed 53.8% similarity and accuracy.

**Table 3:** Similarity and accuracy of 27 items’ zone allocation.

Items	Similarity (%)	Accuracy (%)
Pulse oximeter	100.0	100.0
Levin tube	99.0	99.0
Central line	99.0	99.0
Urine bag	99.0	99.0
Patient bed	99.0	99.0
Blood pressure cuff	98.1	98.1
Bed rail	97.1	97.1
Linen	97.1	97.1
Thermometer	95.2	95.2
Bedside table	91.3	91.3
Stethoscope	90.4	90.4
Telephone fixed beside computer	90.4	90.4
Pole	89.4	89.4
Computer	89.4	89.4
Keyboard	88.5	88.5
CRRT	86.5	86.5
Patient monitor	85.6	85.6
Ventilator monitor	83.7	83.7
Tourniquet	83.7	83.7
Infusion pump	83.7	83.7
Glucometer	73.1	21.2
Flashlight	72.1	72.1
Trolley	70.2	70.2
Sink	66.3	66.3
Another patient	57.7	57.7
Patient cart	53.8	53.8
Curtain	48.1	46.2

CRRT, continuous renal replacement therapy

None of the participants achieved a 100% accuracy. However, participants with a master’s degree or higher showed significantly higher similarity (*p* = .044) or accuracy (*p* = .033). Furthermore, participants with prior knowledge of the patient zone concept demonstrated significantly higher accuracy (*p* = .009; Table 4).

**Table 4:** Similarity and accuracy according to the characteristics of the participants.

Characteristic	Similarity		Accuracy	
	Mean ± SD	*W* or χ^2^ or *r* (*p*)	Mean ± SD	*W* or χ^2^ or *r* (*p*)
**Age**				
**< 25**	83.64 ± 9.50	χ^2^ = 2.62 (0.455)	80.92 ± 9.41	χ^2^ = 5.77 (0.123)
**25 ≤ ~ < 30**	83.70 ± 11.60		81.42 ± 11.72	
**30 ≤ ~ < 35**	85.36 ± 12.86		85.20 ± 12.98	
**≥ 35**	88.41 ± 5.39		85.45 ± 5.67	
**Gender**				
**Male**	89.64 ± 12.38	*W* = 168 (0.223)	87.42 ± 10.33	*W* = 162 (0.191)
**Female**	84.49 ± 10.78		82.51 ± 11.03	
**Total clinical experience (years)**				
**< 1**	80.88 ± 13.32	χ^2^ = 1.81 (0.772)	79.03 ± 11.14	χ^2^ = 2.78 (0.595)
**1 ≤ ~ < 3**	84.46 ± 10.62		81.39 ± 11.62	
**3 ≤ ~ < 5**	83.53 ± 11.48		82.98 ± 11.29	
**5 ≤ ~ < 10**	84.71 ± 13.23		83.59 ± 13.11	
**≥ 10**	87.61 ± 6.38		85.02 ± 6.41	
**ICU experience (years)**				
**< 1**	80.88 ± 13.32	χ^2^ = 2.50 (0.644)	79.03 ± 11.14	χ^2^ = 1.81 (0.771)
**1 ≤ ~ < 3**	84.71 ± 10.30		82.18 ± 11.51	
**3 ≤ ~ < 5**	83.16 ± 11.29		82.43 ± 10.86	
**5 ≤ ~ < 10**	84.67 ± 13.83		83.08 ± 13.64	
**≥ 10**	87.73 ± 6.53		85.01 ± 6.58	
**Department**				
**MICU1**	86.43 ± 4.40	χ^2^ = 8.79 (0.117)	85.61 ± 5.37	χ^2^ = 4.58 (0.470)
**SICU1**	88.23 ± 6.79		85.20 ± 8.91	
**NRICU**	85.36 ± 13.65		81.01 ± 13.55	
**NSICU**	90.29 ± 4.82		87.98 ± 3.83	
**PICU1**	79.77 ± 15.71		80.26 ± 16.49	
**PICU2**	82.90 ± 9.86		81.24 ± 9.58	
**Level of Education**				
**≤ Bachelor**	84.15 ± 11.14	*W* = 255.5 (0.044)	82.12 ± 11.23	*W* = 245 (0.033)
**≥ Master**	90.96 ± 3.26		89.31 ± 4.70	
**Prior knowledge of patient zone concept**				
**No**	78.46 ± 13.45	*W* = 330.5 (0.058)	74.09 ± 14.26	*W* = 265.5 (0.009)
**Yes**	85.60 ± 10.33		83.87 ± 10.17	
**Learning route for patient zone concept**				
**School only**	86.31 ± 8.55	χ^2^ = 0.41 (0.938)	84.09 ± 9.24	χ^2^ = 2.43 (0.487)
**Hospital only**	85.76 ± 10.75		84.41 ± 10.60	
**School & Hospital**	84.21 ± 10.32		81.50 ± 9.38	
**Hospital & Others**	88.90		81.50	

ICU, intensive care unit; SD, standard deviation; MICU, medical intensive care unit; SICU, surgical intensive care unit; NRICU, neurological intensive care unit; NSICU, neurosurgical intensive care unit; PICU, paediatric intensive care unit

### Nurses’ perceptions of items’ zone allocation

Forty-four participants provided insights into the factors influencing the categorisation of items into different zones depending on various scenarios, such as whether the item was located within a private room or in the open ICU space, designated for exclusive use by a single patient or shared among multiple patients, and came into direct contact with the patient. In particular, portable items can be categorised differently because they often straddle both zones contingent on their current use or storage state. Furthermore, some participants indicated that items such as a patient cart could potentially be considered the healthcare zone if they functioned as a station for medication preparation.

Of the 84 participants who responded to the criteria for categorising item zones, a common perspective emerged. Items in direct contact with patients were predominantly assigned to the patient zone. Additionally, the participants considered the item’s proximity to the patient, potential for colonisation with patient flora, and need for disinfection, hand hygiene, or the use of personal protective equipment (PPE) when handling the item.

The challenges associated with item zone categorisation during routine tasks among the 48 respondents arose from a range of factors: confined spaces between the items, ambiguous boundaries, frequent relocation of portable items between usage and storage, ambiguous moments for hand hygiene, disinfection, or PPE use, and inconsistent standards across the hospital. While some individuals struggled with distinguishing zones within private rooms, others mentioned that the distinction was more difficult in open shared spaces. Moreover, items not directly in contact with patients but frequently handled by HCP presented their set of challenges.

To address these challenges, participants’ suggestions from 31 respondents included the formulation of clear criteria supported by evidence-based guidelines and the provision of education for HCP. Practical solutions involve implementing visible spatial divisions using stickers, labels, and delineating lines. Furthermore, their suggested solutions include allocating dedicated equipment and expanding space.

## Discussion

This study demonstrated that ICU nurses had a limited perception of distinguishing between the patient and healthcare zones. This study also found that the participants considered a range of factors beyond mere proximity to the patient, making the determination of the patient zone more intricate than its definition. ICU nurses may encounter challenges in distinguishing between zones based on criteria such as patient contact, room type, distance, cleanliness, and item portability.

The top eight items, all categorised within the patient zone, which maintained constant contact with patients and rarely left the patient’s bed area in ICUs, showed over 97% similarity and accuracy. Given that patient contact was a commonly mentioned criterion among participants, classifying these items is relatively straightforward. Among these items, the central line, urine bag, bed rail, and linen were also categorised into the patient zone with high (100%) similarity in a previous study [24]. However, the blood pressure cuff showed only 70% similarity and 30% accuracy, as IPC experts allocated it to the healthcare zone in a previous study [24]. This might be due to more occasions for sharing a blood pressure cuff in general medical wards in that previous study compared designated single patient use of a blood pressure cuff in our ICUs, emphasising the importance of considering the context of item use across different healthcare settings when applying zone concepts.

Notably, the curtain had the lowest similarity (48.1%) and accuracy (46.2%), reflecting significant variation in our participants’ perceptions. Some participants argued that it should belong to the patient zone if in an open area but to the healthcare zone if in a private room, whereas others deemed everything within a private room to be the patient zone. However, more than 88% of the participants classified the telephone, computer, and keyboard within a private room as belonging to the healthcare zone, suggesting that most participants did not view everything within a private room as part of the patient zone. Meanwhile, some participants found it more challenging to categorise items in open areas, emphasising the complexity that arises when physical boundaries are absent.

Participants considered the distance from patients when distinguishing between zones, which aligns with the definition of the patient zone as the immediate surroundings of the patient [17]. Consequently, participants also considered the positions of adjacent items, revealing the inherent complexity of categorising zones within the ICU. These participants’ perceptions underscore how the placement of nearby items can affect zone allocation, highlighting the intricate interplay between items in patient and healthcare zones. This complexity suggests that patient proximity alone may be insufficient to distinguish between zones clearly. This may explain why some participants encountered difficulties when patient and healthcare zone items coexisted within the confined ICU spaces. Therefore, detailed criteria with explanations clarifying zone definitions must be developed to address the challenges of HCP.

In our study, items potentially contaminated by patient flora were typically categorised as the patient zone, whereas other items kept clean or sterile were generally considered the healthcare zone. This perspective aligns with the notion that the patient zone is susceptible to colonisation by flora, as supported by Sax et al. [17]. However, criticism exists regarding the idea of treating the area outside the patient zone as a single homogeneous zone because this oversimplifies the complexity of the healthcare environment [19]. In this study, we found a conflict between judgements based on the distance and cleanliness. For instance, concerning the patient cart, only a 7.6% difference was observed in the classification into the two zones, making it the second lowest similarity score after the curtain. Despite its proximity to the patient, some participants suggested that if the patient cart was used for medication preparation which required cleanliness, it should be categorised as a healthcare zone. Participants often categorised zones based on whether an item could be touched without necessitating hand hygiene after patient contact or wearing a gown after contact with an isolated patient. Disinfection requirements for an item were also identified as criteria in a previous study [24]. Nevertheless, the concept of the five hand hygiene moments primarily guides the timing of item disinfection rather than the inverse inference that the need for disinfection determines the item’s zone [24]. Therefore, if cleanliness is not a suitable consideration, HCP education must address and correct misconceptions.

The difficulty in categorising zones ultimately leads to challenges in determining appropriate moments for hand hygiene [22], which can lead to patient harm [25]. The discrepancies observed between participants and IPC practitioners may result in the insufficient separation of microorganisms in clinical practice [24]. When reviewing items posing challenges for hand hygiene or item disinfection owing to zone differentiation, many were portable. Glucometer was the prime example of such an item and was misclassified as the healthcare zone by most participants (73.1%). In a previous study, mobile items posed the most significant allocation challenge because of their mobility across zones [24]. When determining zones for portable items is challenging, the risk of HAIs transmission increases. In a study monitoring 27 HCP over 39 hours to analyse behaviours with infection transmission risks, the third most common error out of 8 was the misplacement of items [28]. This resulted in contaminated items sometimes getting mixed with clean items and patient room items ending up in sterile areas [28]. In another study, low disinfection compliance was observed for items such as medication scanners that frequently moved in and out of the patient rooms [29]. As our two participants suggested for pupilometers, storing dedicated patient items within their designated patient zone could effectively reduce contamination risk.

To tackle the challenges associated with zone classification, it is imperative to establish well-defined criteria for patient and healthcare zones, consider space redesigns, and implement educational programs focusing on zone differentiation. In line with the study by Smith et al., where hand contact events were observed and the patient zone was redefined in an operating room, IPC practitioners and frontline nurses should collaborate and reach a consensus on the definition of patient and healthcare zones within their respective departments [23]. Participants’ suggestions to mark zones with stickers, tape, or lines on the floor align with recommendations from previous studies to improve participants’ comprehension and mitigate contamination risk [25, 30]. Zone demarcation clarifies which item belongs to each zone and serves as a direct visual cue for HCP to perform hand hygiene [31]. As participants with higher education levels or prior knowledge displayed better accuracy, future research could explore the potential effectiveness of training programs. Such programs might enhance HCP’s understanding of patient and healthcare zones, consequently improving their ability to determine the timing of hand hygiene and disinfection.

Our study has several limitations. Our study’s response rate was relatively low (46.2%), likely due to nurses’ fatigue caused by the exhaustive additional work required for the hospital’s accreditation preparation during the data collection period, leaving little time or energy to complete our extensive scenario-based survey. Despite these barriers, nurses who volunteered may have possessed more interest in the patient zone concept, which could have influenced the study outcomes. As 88.5% of the participants had prior knowledge about the zones, nurses unfamiliar with the two zone concepts might have avoided participating in this study. While the nursing care scenarios were refined through expert validation, predicting item difficulty was inherently limited. Furthermore, the uneven distribution of items between zones in the scenarios, with only seven in the healthcare zone, might have introduced biased results. Future studies should include a broader spectrum of items and verified scenarios to obtain more precise and comprehensive results.

Given that this study was conducted in six ICUs at a single hospital in Korea, replicating it across diverse healthcare settings and among various HCP would provide more comprehensive insights, particularly for infection control practices. The use of detailed scenarios and illustrations in this study allowed nurses from different settings to gain a consistent understanding of the situations, enhancing the potential to generalise the findings to other hospital contexts or regions. Since item placement can significantly influence zone classification, hospitals should assess nurses’ perceptions within the context of their specific environment and item arrangements. This approach will enable the development of optimised environmental management recommendations tailored to each hospital’s unique setup, ultimately contributing to more effective infection control strategies.

## Conclusions

In our study, ICU nurses showed higher accuracy (82.7%) and similarity (84.7%) in distinguishing between patient and healthcare zones compared to a previous study conducted in a general medical ward, which reported 67.7% accuracy and 76.6% similarity [24]. Unlike the previous study using item cards, we adopted three nursing care scenarios with detailed illustrations showing exact item locations to examine ICU nurses’ perceptions of the two zones, which is a notable strength of our approach. Consequently, our study revealed that participants could exhibit variations in their zones, reconfirming that the patient zone is not a geographically static area but rather varies depending on the setting and type of care delivered [32]. This observation highlights the need for further research to establish greater clarity and consensus regarding patient and healthcare zones to enhance infection control practices among HCP.

## Supporting information

S1 File The English version of the questionnaire. (DOCX)

## References

[1] European Centre for Disease Prevention and Control. Point prevalence survey of healthcare-associated infections and antimicrobial use in European acute care hospitals 2016–2017. Solna; 2023.

[2] Donskey CJ. Beyond high-touch surfaces: Portable equipment and floors as potential sources of transmission of health care–associated pathogens. Am J Infect Control. 2019;47:A90–A5. doi: 10.1016/j.ajic.2019.03.017 31146857

[3] Weber DJ, Rutala WA. Understanding and Preventing Transmission of Healthcare-Associated Pathogens Due to the Contaminated Hospital Environment. Infect Control Hosp Epidemiol. 2013;34(5):449–52. doi: 10.1086/670223 23571359

[4] Otter JA, Yezli S, Salkeld JA, French GL. Evidence that contaminated surfaces contribute to the transmission of hospital pathogens and an overview of strategies to address contaminated surfaces in hospital settings. Am J Infect Control. 2013;41(5 Suppl):S6–11. 23622751 10.1016/j.ajic.2012.12.004

[5] Alhmidi H, Cadnum JL, Jencson AL, Gweder AA, Donskey CJ. Sharing is not always a good thing: Use of a DNA marker to investigate the potential for ward-to-ward dissemination of healthcare-associated pathogens. Infect Control Hosp Epidemiol. 2019;40(2):214–6. doi: 10.1017/ice.2018.320 30522541

[6] Alhmidi H, Cadnum JL, Koganti S, Jencson AL, Rutter JD, Bonomo RA, et al. Shedding of methicillin-resistant Staphylococcus aureus by colonized patients during procedures and patient care activities. Infect Control Hosp Epidemiol. 2019;40(3):328–32. doi: 10.1017/ice.2018.342 30777587

[7] Musuuza JS, Hundt AS, Carayon P, Christensen K, Ngam C, Haun N, et al. Implementation of a Clostridioides difficile prevention bundle: Understanding common, unique, and conflicting work system barriers and facilitators for subprocess design. Infect Control Hosp Epidemiol. 2019;40(8):880–8. doi: 10.1017/ice.2019.150 31190669 PMC7212912

[8] Clack L, Passerini S, Wolfensberger A, Sax H, Manser T. Frequency and Nature of Infectious Risk Moments During Acute Care Based on the INFORM Structured Classification Taxonomy. Infect Control Hosp Epidemiol. 2018;39(3):272–9. doi: 10.1017/ice.2017.326 29498339

[9] Ahn JY, An S, Sohn Y, Cho Y, Hyun JH, Baek YJ, et al. Environmental contamination in the isolation rooms of COVID-19 patients with severe pneumonia requiring mechanical ventilation or high-flow oxygen therapy. J Hosp Infect. 2020;106(3):570–6. doi: 10.1016/j.jhin.2020.08.014 32828864 PMC7441047

[10] Chemaly RF, Simmons S, Dale C Jr., Ghantoji SS, Rodriguez M, Gubb J, et al. The role of the healthcare environment in the spread of multidrug-resistant organisms: update on current best practices for containment. Ther Adv Infect Dis. 2014;2(3–4):79–90. doi: 10.1177/2049936114543287 25469234 PMC4250270

[11] Hartmann B, Benson M, Junger A, Quinzio L, Röhrig R, Fengler B, et al. Computer Keyboard and Mouse as a Reservoir of Pathogens in an Intensive Care Unit. J Clin Monit Comput. 2004;18(1):7–12. doi: 10.1023/b:jocm.0000025279.27084.39 15139578

[12] Kanamori H, Rutala WA, Weber DJ. The Role of Patient Care Items as a Fomite in Healthcare-Associated Outbreaks and Infection Prevention. Clin Infect Dis. 2017;65(8):1412–9. doi: 10.1093/cid/cix462 28520859

[13] Phan LT, Sweeney DM, Maita D, Moritz DC, Bleasdale SC, Jones RM. Respiratory viruses in the patient environment. Infect Control Hosp Epidemiol. 2020;41(3):259–66. doi: 10.1017/ice.2019.299 32043434

[14] Shimohata T, Mawatari K, Uebanso T, Honjo A, Tsunedomi A, Hatayama S, et al. Bacterial Contamination of Hemodialysis Devices in Hospital Dialysis Wards. J Med Invest. 2019;66(1.2):148–52. doi: 10.2152/jmi.66.148 31064928

[15] Thompson ND, Hellinger WC, Kay RS, Cohen L, Ragan P, Voss RA, et al. Healthcare-associated hepatitis C virus transmission among patients in an abdominal organ transplant center. Transpl Infect Dis. 2009;11(4):324–9. doi: 10.1111/j.1399-3062.2009.00406.x 19497073

[16] Wright EW, Marvel JH, Neubrander J, DesMarteau K. Analysis and Recommendations for Reducing Risks of Patient Crosscontaminations Via Noncritical Medical Devices. The Health Care Manager. 2014;33(3):205–13. doi: 10.1097/HCM.0000000000000015 25068874

[17] Sax H, Allegranzi B, Uçkay I, Larson E, Boyce J, Pittet D. My five moments for hand hygiene: a user-centred design approach to understand, train, monitor and report hand hygiene. J Hosp Infect. 2007;67(1):9–21. doi: 10.1016/j.jhin.2007.06.004 17719685

[18] World Health Organization. WHO Guidelines on Hand Hygiene in Health Care. 2009.

[19] Gould D, Purssell E, Jeanes A, Drey N, Chudleigh J, McKnight J. The problem with ’My Five Moments for Hand Hygiene’. BMJ Qual Saf. 2022;31(4):322–6. doi: 10.1136/bmjqs-2020-011911 34261814 PMC8938669

[20] Chou DT, Achan P, Ramachandran M. The World Health Organization ‘5 moments of hand hygiene’: the scientific foundation. J Bone Joint Surg Br. 2012;94(4):441–5. doi: 10.1302/0301-620X.94B4.27772 22434456

[21] Salmon S, McLaws ML. Environmental challenges of identifying a patient zone and the healthcare zone in a crowded Vietnamese hospital. J Hosp Infect. 2015;91(1):45–52. doi: 10.1016/j.jhin.2015.04.020 26076809

[22] Jung E, Ha YK, Park N, Kim HH. Survey of ICU Nurses’ Knowledge of the Specific Moments of Hand Hygiene. Journal of Korean Critical Care Nursing. 2017;10(2):56–70.

[23] Smith F, Lee K, Binnie-McLeod E, Higgins M, Irvine E, Henderson A, et al. Identifying the World Health Organization’s fifth moment for hand hygiene: Infection prevention in the operating room. Journal of Infection Prevention. 2020;21(1):28–34. doi: 10.1177/1757177419879996 32030101 PMC6978567

[24] Bogdanovic J, Petralito S, Passerini S, Sax H, Manser T, Clack L. Exploring healthcare providers’ mental models of the infection prevention “patient zone”—a concept mapping study. Antimicrobial Resistance and Infection Control. 2019;8:138. doi: 10.1186/s13756-019-0593-4 31428315 PMC6694681

[25] Sax H, Clack L. Mental models: a basic concept for human factors design in infection prevention. J Hosp Infect. 2015;89(4):335–9. doi: 10.1016/j.jhin.2014.12.008 25676111

[26] Siegel JD, Rhinehart E, Jackson M, Chiarello L, Healthcare Infection Control Practices Advisory Committee. Management of Multidrug-Resistant Organisms In Healthcare Settings, 2006. In: Prevention CfDCa, editor. 2017 ed2006. 10.1016/j.ajic.2007.10.006 18068814

[27] English KM, Langley JM, McGeer A, Hupert N, Tellier R, Henry B, et al. Contact among healthcare workers in the hospital setting: Developing the evidence base for innovative approaches to infection control. BMC Infect Dis. 2018;18(1). doi: 10.1186/s12879-018-3093-x 29665775 PMC5905140

[28] Lindberg M, Lindberg M, Skytt B. Risk behaviours for organism transmission in health care delivery-A two month unstructured observational study. Int J Nurs Stud. 2017;70:38–45. doi: 10.1016/j.ijnurstu.2017.02.016 28231441

[29] Ngam C, Schoofs Hundt A, Haun N, Carayon P, Stevens L, Safdar N. Barriers and facilitators to Clostridium difficile infection prevention: A nursing perspective. Am J Infect Control. 2017;45(12):1363–8. doi: 10.1016/j.ajic.2017.07.009 28939012

[30] Herlihey TA, Gelmi S, Cafazzo JA, Hall TNT. The Impact of Environmental Design on Doffing Personal Protective Equipment in a Healthcare Environment: A Formative Human Factors Trial. Infect Control Hosp Epidemiol. 2017;38(6):712–7. doi: 10.1017/ice.2017.68 28460655

[31] Jansen SJ, Muller BJ, Cramer SJE, Te Pas AB, Lopriore E, Bekker V. Developing a design-based concept to improve hand hygiene in the neonatal intensive care unit. Pediatr Res. 2023;94(2):450–7. doi: 10.1038/s41390-023-02482-9 36694024 PMC10382316

[32] World Health Organization, W. H. O. Patient Safety. Hand hygiene technical reference manual: to be used by health-care workers, trainers and observers of hand hygiene practices. Geneva: World Health Organization; 2009.

