# Supplementary material for: Perceptions of distinctions between patient and healthcare zones among intensive care unit nurses at a Korean tertiary hospital: A cross-sectional study
Source: PLoS One. 2024 Nov 15;19(11):e0311298. doi: 10.1371/journal.pone.0311298 (PMC11567548; doi:10.1371/journal.pone.0311298)
Supplement: S1 File — (DOCX) [file pone.0311298.s001.docx]

**The English version of the questionnaire**

**[Perceptions of Distinctions Between Patient and Healthcare Zones Among Intensive Care Unit Nurses at a Korean Tertiary Hospital: A Cross-sectional Study]**

**Ⅰ. Scenarios**

Starting from the next page, there are illustrations depicting the locations of 27 items used when a nurse provides care to a patient in the intensive care unit, and three scenarios describing the situations in which each item is used. Please read each scenario and determine whether the numbered items belong to the patient zone or the healthcare zone.

For reference, the concepts of the "patient zone" and the "healthcare zone" have been introduced to geographically visualize key moments for hand hygiene [1]. They are also related to removing contamination from items and the environment around the patient [2]. The definitions for both zones are as follows [1].

| Patient zone | The area immediately surrounding Patient X, colonized with the patient's normal flora of bacteria. Surfaces directly in contact with the patient and those frequently touched by healthcare personnel during medical procedures involving the patient are also included. |
| --- | --- |
| Healthcare zone | All surfaces outside the patient zone of Patient X, including other patients, the patient zones of other patients, and the healthcare facility environment. |

To prevent the transmission of microorganisms from the patient zone to the healthcare zone [1] and, conversely, to prevent the entry of microorganisms from the healthcare zone into the patient zone, it is necessary to remove contamination from the hands of healthcare personnel, as well as items and the environment, when moving in and out of the patient zone [3]. Therefore, to determine the moments requiring hand hygiene and disinfection of items and the environment, it is essential to first distinguish between the patient zone and the healthcare zone. If there are differing perceptions among healthcare personnel regarding the patient zone, moments for hand hygiene may not align, unintentionally leading to the spread of healthcare associated infection [4].

**[Scenario 1:** **Assessing a Patient’s Condition and Measuring Vital Signs]**

Please refer to the illustration below and respond to the questions regarding Scenario 1.


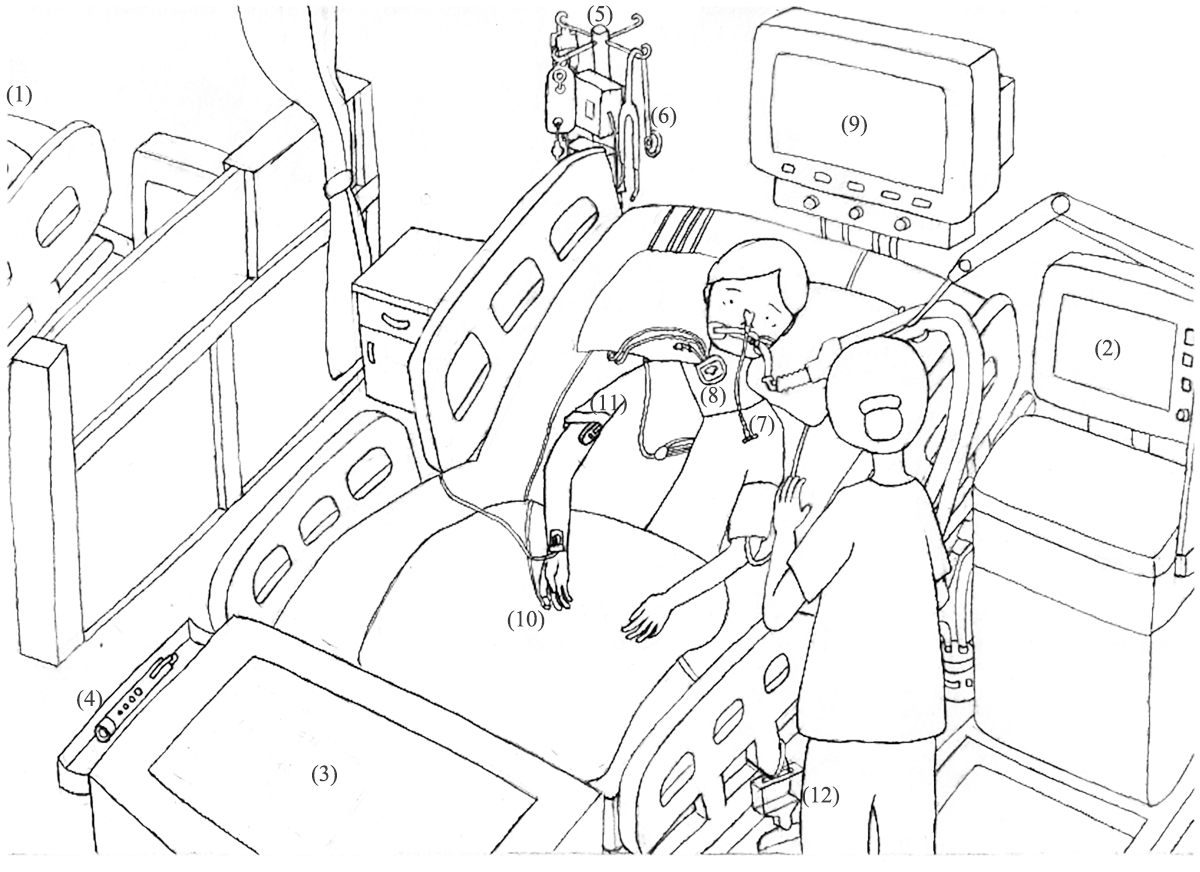


(1) Another Patient, (2) Ventilator Monitor, (3) Patient Cart, (4) Flashlight, (5) Pole, (6) Stethoscope, (7) Levin Tube, (8) Central Line, (9) Patient Monitor, (10) Pulse Oximeter, (11) Thermometer, and (12) Urine Bag

**[Scenario 1:** **Assessing a Patient’s Condition and Measuring Vital Signs]**

Please mark with a 'V' in the box you believe each item belongs to, either the patient zone or the healthcare zone, in the scenario described below. If it is difficult to judge, mark with a 'V' in the box named 'Difficult to determine' and provide the reasons for the difficulty.

| **Scenario** | **Item / Environment** | **Patient zone** | **Healthcare Zone** | **Difficult to determine** | **Reasons for the difficulty** |
| --- | --- | --- | --- | --- | --- |
| • Patient A has been diagnosed with Type 2 respiratory failure and is undergoing mechanical ventilation treatment in the medical intensive care unit.  • Patient A's bed is situated in an open area, separated from the bed of **(1) another patient** by a partition wall.  • Nurse Kim begins her shift and approaches the bedside of her assigned patient, Patient A.  • Nurse Kim presses the **(2) ventilator monitor** button to check the current settings.  • While assessing the patient's consciousness, Nurse Kim retrieves a **(4) flashlight** from the **(3) patient cart** to check for pupillary light reflex.  • Nurse Kim uses the **(6) stethoscope** hanging on the patient's **(5) pole** to auscultate the patient's breath sounds.  • Nurse Kim checks the insertion depth of the **(7) feeding tube** inserted through the patient's nose.  • Nurse Kim assesses the condition and depth of the **(8) central line** insertion site in the right internal jugular vein of the patient.  • Nurse Kim visually checks the blood pressure, heart rate, and oxygen saturation values displayed on the **(9) patient monitor**.  • Due to the unclear oxygen saturation waveform, Nurse Kim secures a proper fit of the **(10) pulse oximeter** on the finger.  • Nurse Kim retrieves a **(11) thermometer** from the drawer containing the patient's personal belongings and places it in the patient's armpit.  • Nurse Kim measures the urine output for the past one hour while draining urine from the **(12) urine bag**. | (1) Another Patient |  |  |  |  |
|  | (2) Ventilator Monitor |  |  |  |  |
|  | (3) Patient Cart |  |  |  |  |
|  | (4) Flashlight |  |  |  |  |
|  | (5) Pole |  |  |  |  |
|  | (6) Stethoscope |  |  |  |  |
|  | (7) Levin Tube |  |  |  |  |
|  | (8) Central Line |  |  |  |  |
|  | (9) Patient Monitor |  |  |  |  |
|  | (10) Pulse Oximeter |  |  |  |  |
|  | (11) Thermometer |  |  |  |  |
|  | (12) Urine Bag |  |  |  |  |

1. If you responded that items (1) to (12) belong to either the patient zone or the healthcare zone, but you think that certain items and environments might belong to a different zone depending on the situation, please specify which items and environments you believe belong to a different zone, and provide the reasons for your response.

**[Scenario 2: Patient Bathing and Measuring Body Weight]**

Please refer to the illustration below and respond to the questions regarding Scenario 2. Note that Patient B's bed is located in a single-patient room.


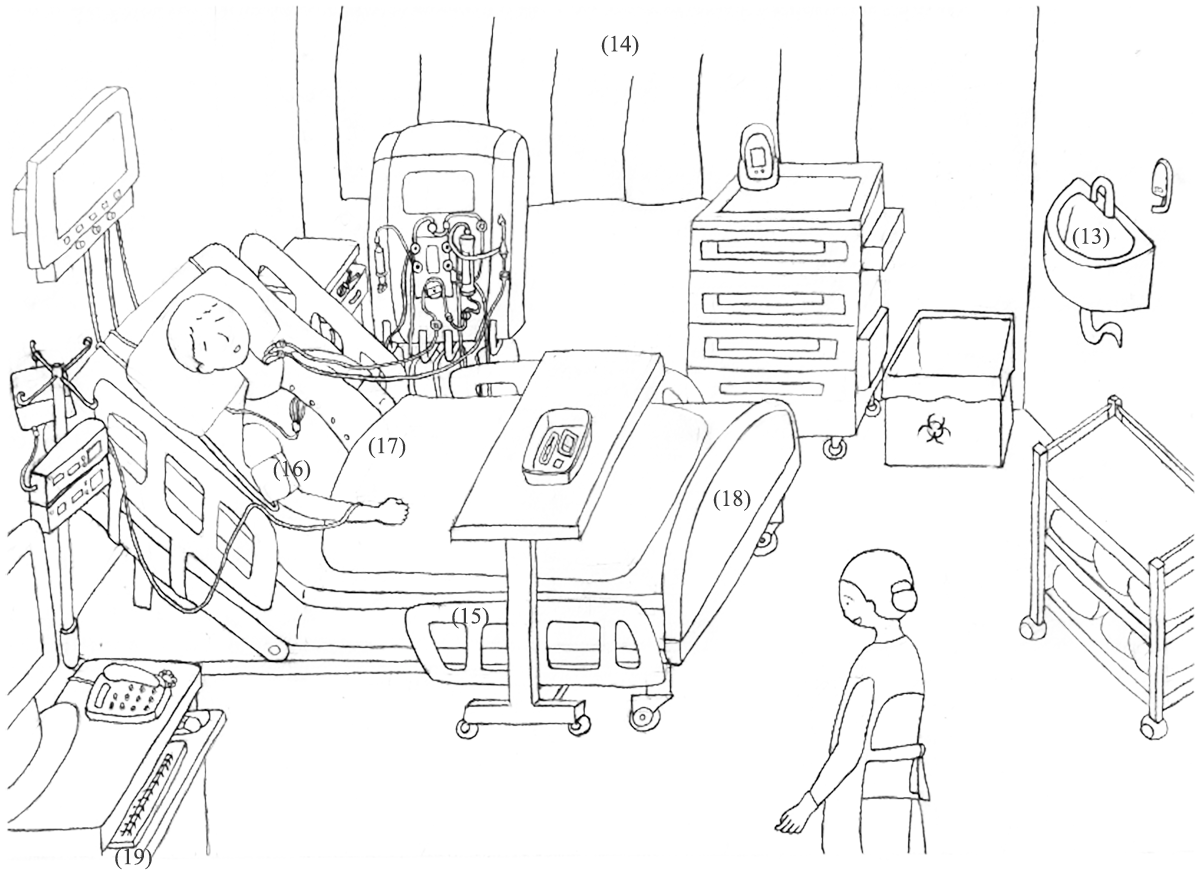


(13) Sink, (14) Curtain, (15) Bed Rail, (16) Blood Pressure Cuff, (17) Linen, (18) Patient Bed, and (19) Keyboard

**[Scenario 2: Patient Bathing and Measuring Body Weight]**

Please mark with a 'V' in the box you believe each item belongs to, either the patient zone or the healthcare zone, in the scenario described below. If it is difficult to judge, mark with a 'V' in the box named 'Difficult to determine' and provide the reasons for the difficulty.

| **Scenario** | **Item / Environment** | **Patient**  **zone** | **Healthcare**  **Zone** | **Difficult to determine** | **Reasons for the difficulty** |
| --- | --- | --- | --- | --- | --- |
| • Nurse Kim's other assigned patient, Patient B, is undergoing continuous renal replacement therapy. Vancomycin-resistant enterococcus was detected in the screening test upon admission, leading to the application of contact precaution.  • Patient B's bed is located in a single-patient room, and the patient cannot move outside the bed.  • During bath time, Nurse Kim wets a towel at the **(13) sink** in front of Patient B's bed.  •. After drawing the **(14) curtain** on the window, Nurse Kim lowers the **(15) bed rail**.  • Nurse Kim loosens the **(16) blood pressure cuff** which was wrapped around the patient's arm.  • Nurse Kim wipes the patient's body with a towel and replaces the used **(17) linens** (gown, sheets, pillowcase) with fresh ones.  • Adjusting the height of the **(18) patient bed** and ensuring that no part touches the floor, Nurse Kim proceeds to measure the patient's weight.  • Nurse Kim tidies up the bed and records the patient's weight in the electronic medical records using the **(19) keyboard**. | (13) Sink |  |  |  |  |
|  | (14) Curtain |  |  |  |  |
|  | (15) Bed Rail |  |  |  |  |
|  | (16) Blood Pressure Cuff |  |  |  |  |
|  | (17) Linen |  |  |  |  |
|  | (18) Patient Bed |  |  |  |  |
|  | (19) Keyboard |  |  |  |  |

2. If you responded that items (13) to (19) belong to either the patient zone or the healthcare zone, but you think that certain items and environments might belong to a different zone depending on the situation, please specify which items and environments you believe belong to a different zone, and provide the reasons for your response.

|  |
| --- |

**[Scenario 3: Measuring Blood Glucose Levels and Administering Medication]**

Please refer to the illustration below and respond to the questions regarding Scenario 3. Note that Patient B in Scenarios 2 and 3 are the same person.


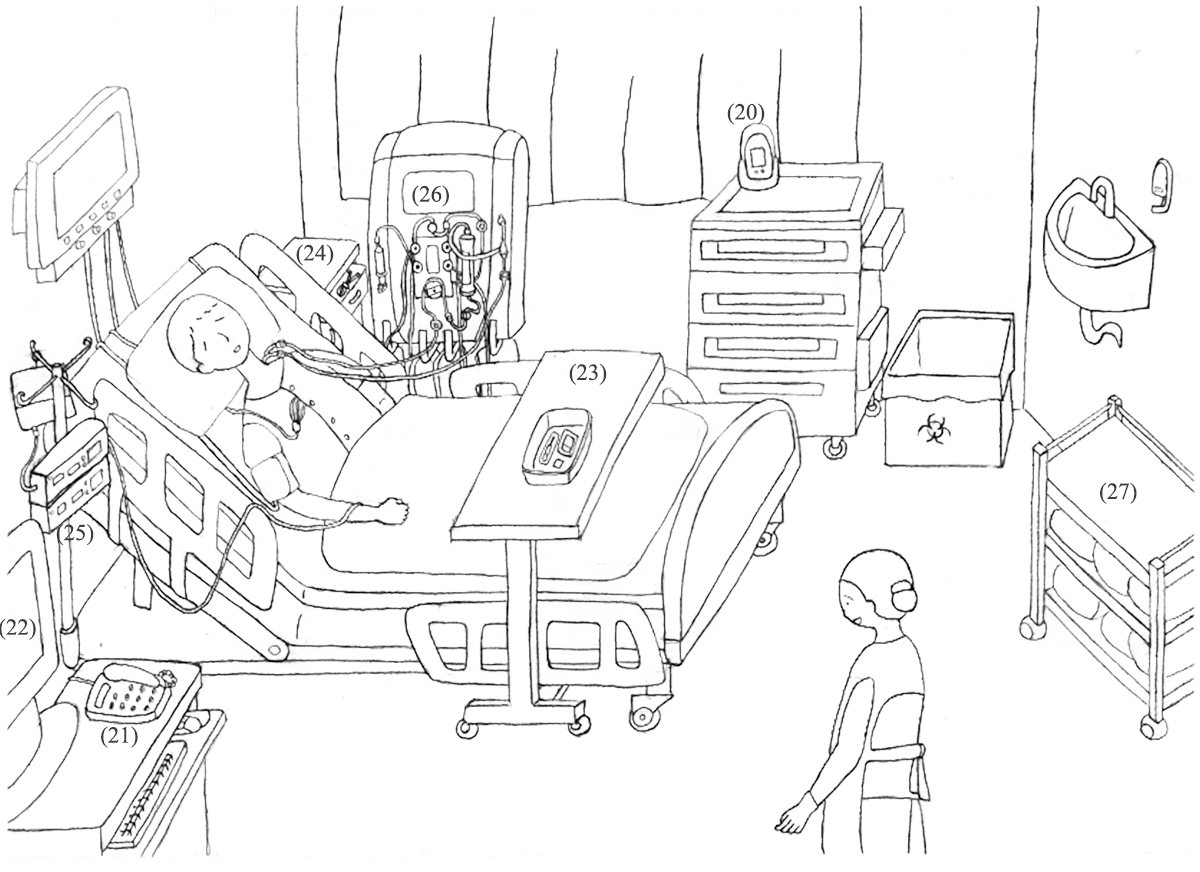


(20) Glucometer, (21) Telephone Fixed Beside Computer, (22) Computer, (23) Bedside Table, (24) Tourniquet, (25) Infusion Pump, (26) Continuous Renal Replacement Therapy Machine, and (27) Trolley

**[Scenario 3: Measuring Blood Glucose Levels and Administering Medication]**

Please mark with a 'V' in the box you believe each item belongs to, either the patient zone or the healthcare zone, in the scenario described below. If it is difficult to judge, mark with a 'V' in the box named 'Difficult to determine' and provide the reasons for the difficulty.

| **Scenario** | **Item / Environment** | **Patient**  **zone** | **Healthcare**  **Zone** | **Difficult to determine** | **Reasons for the difficulty** |
| --- | --- | --- | --- | --- | --- |
| • Patient B, who has diabetes, is currently on fasting due to the possibility of intubation and is receiving 24-hour total parenteral nutrition.  • Nurse Kim takes the **(20) glucometer** from the cart and measures the patient's blood glucose.  • Nurse Kim reports the blood glucose trend to the primary physician using the telephone **(21) fixed beside computer**.  • Nurse Kim verifies the order on the **(22) computer** to administer continuous regular insulin (RI).  • Due to a shortage of intravenous injection routes for administering RI alone, Nurse Kim brings the necessary supplies for securing a new intravenous injection site and places them on the **(23) bedside table**.  **•**After retrieving a **(24) tourniquet** (designated for Patient B) from the drawer containing the patient's personal belongings, Nurse Kim ties it around the patient's arm and secures an IV route in the peripheral vein.  • After preparing the RI solution, Nurse Kim hangs it on the **(25) infusion pump** attached to the patient's bed, sets the infusion rate, and connects it to the patient.  • An alarm sounds indicating an empty dialysis bag in **(26) continuous renal replacement therapy machine**. Nurse Kim retrieves a new dialysis bag designated for Patient B from the **(27) trolley**, replacing the empty one.  • Nurse Kim disposes of the empty dialysis bag in the trash bin inside the patient's room. | (20) Glucometer |  |  |  |  |
|  | (21) Telephone Fixed Beside Computer |  |  |  |  |
|  | (22) Computer |  |  |  |  |
|  | (23) Bedside Table |  |  |  |  |
|  | (24) Tourniquet |  |  |  |  |
|  | (25) Infusion Pump |  |  |  |  |
|  | (26) Continuous Renal Replacement Therapy Machine |  |  |  |  |
|  | (27) Trolley |  |  |  |  |

3. If you responded that items (20) to (27) belong to either the patient zone or the healthcare zone, but you think that certain items and environments might belong to a different zone depending on the situation, please specify which items and environments you believe belong to a different zone, and provide the reasons for your response.

|  |
| --- |

**Ⅱ. Additional Opinions**

4. What criteria do you consider most important when categorising items to the patient zone or the healthcare zone?

|  |
| --- |

5. Please describe any difficulties or challenges you have encountered when distinguishing between the patient zone and the healthcare zone during regular work.

|  |
| --- |

6. If you have any suggestions for addressing the difficulties or challenges mentioned above, please provide them.

|  |
| --- |

7. If you have had difficulty distinguishing between the patient zone and the healthcare zone, leading to confusion when determining the appropriate time for disinfection or hand hygiene, please specify which items caused these challenges. Describe the situations in which these items were used, and if you have any suggestions for improving such situations, please provide them. Feel free to include items not mentioned in the scenarios.

|  |
| --- |

**Ⅲ. General Characteristics**

The following are questions regarding general characteristics. Please provide answers for each question or mark the corresponding items with a checkmark (V).

8. What is your age? _____ years-old.

9. What is your gender? ① Male ② Female

10. How many years and months of total clinical experience do you have?

___ year(s) and ___ month(s)

11. How many years and months of clinical experience in intensive care unit (ICU) do you have?

___ year(s) and ___ month(s)

12. In which department do you work?

① Medical ICU 1 ② Surgical ICU 1 ③ Neurological ICU

④ Neurosurgical ICU ⑤ Paediatric ICU 1 ⑥ Paediatric ICU 2

13. What is your educational background?

① Associate degree ② Bachelor degree ③ Master’s degree ④ Doctor degree

14. Were you familiar with the concepts of the patient and healthcare zones before participating in this study?

① No 🡪 End of the survey.

② Yes 🡪 Go to the question number 14-1.

14-1. Through which channel did you become acquainted with the concepts of the patient and healthcare zones?

1. College education ② Hospital training ③ Others: ___________________

**- End. Thank you for participating in the survey. -**

**References**

1. Sax H, Allegranzi B, Uçkay I, Larson E, Boyce J, Pittet D: **My five moments for hand hygiene: a user-centred design approach to understand, train, monitor and report hand hygiene**. *J Hosp Infect* 2007, **67**(1):9-21.

2. Bogdanovic J, Petralito S, Passerini S, Sax H, Manser T, Clack L: **Exploring healthcare providers’ mental models of the infection prevention “patient zone” - a concept mapping study**. *Antimicrobial Resistance and Infection Control* 2019, **8**:138.

3. World Health Organization: **WHO Guidelines on Hand Hygiene in Health Care**. In*.*; 2009.

4. Sax H, Clack L: **Mental models: a basic concept for human factors design in infection prevention**. *J Hosp Infect* 2015, **89**(4):335-339.
