# Supplementary material for: Perceptions of distinctions between patient and healthcare zones among intensive care unit nurses at a Korean tertiary hospital: A cross-sectional study
Source: PLoS One. 2024 Nov 15;19(11):e0311298. doi: 10.1371/journal.pone.0311298 (PMC11567548; doi:10.1371/journal.pone.0311298)
Supplement: Attachment — Submitted filename: Manuscript Review_01.docx [file pone.0311298.s002.docx]

**Manuscript Review**

**Reviewer’s Information**

| E-Mail: | [dtienyi@gmail.com](mailto:dtienyi@gmail.com) |
| --- | --- |
| Title: | Mr. |
| First Name: | Tienyi |
| Last Name: | Daniel |
| Affiliation: | Testimony College of Health and Allied Sciences(TECOHAS) |
| Country: | Tanzania |
| Specialization: | Lecturer |

**Manuscript Information**

| Journal Name: | PLOS ONE |
| --- | --- |
| Manuscript Number: | PONE-D-24-25721 |
| Manuscript Title: | Perceptions of distinctions between patient and healthcare zones among intensive care unit nurses at a Korean tertiary hospital: a cross-sectional study |
| Date Received from Journal: | **09/07/2024** |
| Date to Send Review Report: | **15/07/2024** |

**Reviewer’s Blind Review Comments to Author**

| 5. | Abstract: | I would suggest expanding this part slightly to include more details, for example of **Background section** should be comprehensive highlighting clear and concise overview of the research, **Methodology** section should include approach used, number of participants recruited. I also noticed on factors considered by patients on this statement ‘***Participants considered factors beyond proximity to the patient, including patient contact, room type, distance, cleanliness, and item portability, in distinguishing between the two zones***’ there is no  the statistical results (e.g., chi-square values, p-values) that support the relationships between the identified factors. |
| --- | --- | --- |
| 6. | Introduction: | The introduction section does not provide a **clear rationale or objectives** for the current study. While the authors have presented a comprehensive review of the existing literature, the introduction **lacks a clear statement of the research problem or gap that this study aims to address**. It is crucial to explicitly articulate the research objectives at the last paragraph of introduction, as this sets the foundation for the entire study and helps the reader understand the significance and contributions of the work. |
| 8. | Methodology: | The methodology section is generally well-described, with details provided on the study design, participant recruitment, and data collection procedures. However, I noticed that the manuscript does not include information on the **number of participants** recruited for the study. This is an important detail that should be included, as it allows the reader to assess the statistical power and generalizability of the findings. Also on **page 6**  where there is description of an illustration that part should be on ***Supplemental material*** not methodology part. |
| 9. | Results: | The results section is particularly well-written and presented. The authors have done an excellent job of organizing the findings in a clear and coherent manner, using appropriate statistical reporting. |
| 10. | Discussions: | Authors have provided a comprehensive, and reasoned interpretation of the results. |
| 11. | Conclusions: | Meaningful and well written according to the study findings. |
| 12. | References / Bibliography: | The authors used numbered-reference format which is very useful and easier in reading and follow up. |
| 16. | Reviewer’s Decision Comment: | **Manuscripts require minor corrections.** |

**Recommandation**

| Accept As Is: |  |
| --- | --- |
| Requires Minor Corrections: | **✓** |
| Requires Moderate Revision: |  |
| Requires Major Revision: |  |
| Submit To Another Publication Such As: |  |
| Rejection (Please provide reasons) |  |
